# Variability in Language used on Social Media prior to Hospital Visits

**DOI:** 10.1038/s41598-020-60750-8

**Published:** 2020-03-12

**Authors:** Sharath Chandra Guntuku, H. Andrew Schwartz, Adarsh Kashyap, Jessica S. Gaulton, Daniel C. Stokes, David A. Asch, Lyle H. Ungar, Raina M. Merchant

**Affiliations:** 1 University of Pennsylvania, Philadelphia, PA USA; 2 Stony Brook University, NY, USA; 3 Children’s Hospital of Pennsylvania, Philadelphia, PA USA; 4 Cpl Michael J Crescenz VA Medical Center, Philadelphia, PA USA

**Keywords:** Epidemiology, Computer science

## Abstract

Forecasting healthcare utilization has the potential to anticipate care needs, either accelerating needed care or redirecting patients toward care most appropriate to their needs. While prior research has utilized clinical information to forecast readmissions, analyzing digital footprints from social media can inform our understanding of individuals’ behaviors, thoughts, and motivations preceding a healthcare visit. We evaluate how language patterns on social media change prior to emergency department (ED) visits and inpatient hospital admissions in this case-crossover study of adult patients visiting a large urban academic hospital system who consented to share access to their history of Facebook statuses and electronic medical records. An ensemble machine learning model forecasted ED visits and inpatient admissions with out-of-sample cross-validated AUCs of 0.64 and 0.70 respectively. Prior to an ED visit, there was a significant increase in depressed language (Cohen’s d = 0.238), and a decrease in informal language (d = 0.345). Facebook posts prior to an inpatient admission showed significant increase in expressions of somatic pain (d = 0.267) and decrease in extraverted/social language (d = 0.357). These results are a first step in developing methods to utilize user-generated content to characterize patient care-seeking context which could ultimately enable better allocation of resources and potentially early interventions to reduce unplanned visits.

## Introduction

There are approximately 137 million emergency department (ED) visits and 36 million inpatient admissions annually in the United States (US). Forecasting healthcare utilization has the potential to anticipate care needs, either accelerating needed care or redirecting patients toward care most appropriate to their needs. The behavioral traces people leave in their social media and digital activities embed clues about health and health care behaviors, creating an opportunity for such forecasting.

Indeed, many Internet users spend nearly 10% of waking time each day using social media platforms like Facebook, Twitter, Instagram, and Snapchat, generating or consuming content^1^. Approximately 15–25% of this content is health-related^2^. As a result, social media now provides a view into an enormous sector of patients’ health that was previously unobservable. Statistical and machine learning language processing techniques have been used to relate social media language use to a wide variety of health-related outcomes including mood^3^ and mental health attributes such as depression^4^, suicidal ideation^5,6^, loneliness^7^, and post-traumatic stress disorder^8^.

Social media sources provide an opportunity to evaluate data at the individual level, and their accessibility supports studies to test their predictive power and to generate insights about what underlies observed associations between people’s communications and their health. For example, depressed individuals use more first-person singular pronouns suggesting higher self-focus^9,10^. Natural language processing and machine learning automate the analysis of posts that would have been too numerous to evaluate manually. These tools have revealed their value, with studies showing that Facebook and Twitter posts can be used to predict mental health diagnoses and outcomes. To date, only a few studies have evaluated the potential contribution of digital data in studying healthcare utilization at the individual level^11^.

We sought to develop a machine learning framework to evaluate if posts on social media change before an ED visit or inpatient hospitalization. We aimed to answer two questions: 1) Can social media language forecast healthcare utilization? 2) Which specific linguistic characteristics in social media posts change before a hospital visit?

## Results

### Participants

**Figure 1:**
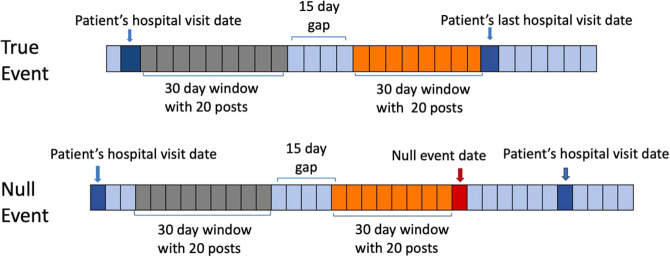
Defining time periods prior true (hospital visits) and null (random time point) events. Figure shows the time periods before a hospital visit and random time points from which changes in linguistic features were calculated. Dark blue points are hospital visits (true event). Red point is a random time point (null event). Grey and Orange windows are 30 day periods, separated by a 15 day window, prior to true and null events.

Of the 5,401 patients seeking care at an urban academic hospital who consented to share their Facebook posts along with their electronic medical record (EMR) data, 2915 individuals had posts that we obtained. We used the case-crossover design^12^ to distinguish change in users’ social media language features before a hospital visit compared to the change prior to any random time point (Fig. 1). 419 patients with an emergency visit without inpatient admission and 167 with inpatient admissions met the eligibility criteria. Of 419 included in the analysis for emergency visits, 84% were African American, 89% were women, and the median age was 28. Of 167 included in the analysis for inpatient visits, 86% were African American and 91% were women, with a median age of 30 (Table 1). Of 167 patients who were admitted to inpatient services, 117 initially received care in the ED. The primary ED visits were for unspecified abdominal pain, chest pain, and headache; and the primary inpatient admissions were pregnancy-related, sickle cell disease with crisis, and shortness of breath. We compared the age, gender, and race of the participants who were included in this analysis to those who were not and found no significant difference.

**Table 1:** Demographic characteristics of patients included in emergency and inpatient visit analysis. Every patient is their own control.

Emergency	
	N
**Total Participants**	419
**Race**	
African American	352 (84%)
White	53 (13%)
Other	14 (3%)
**Females**	374 (89%)
**Age range (yrs)**	[19–81]
**Age median (yrs)**	28
**Inpatient**	
**Total Participants**	167
**Race**	
African American	143 (86%)
White	21 (13%)
Other	3 (1%)
**Females**	152 (91%)
**Age range (yrs)**	[19–81]
**Age median (yrs)**	30

### Predicting hospital visits using changes in language prior to the visit

**Figure 2:**
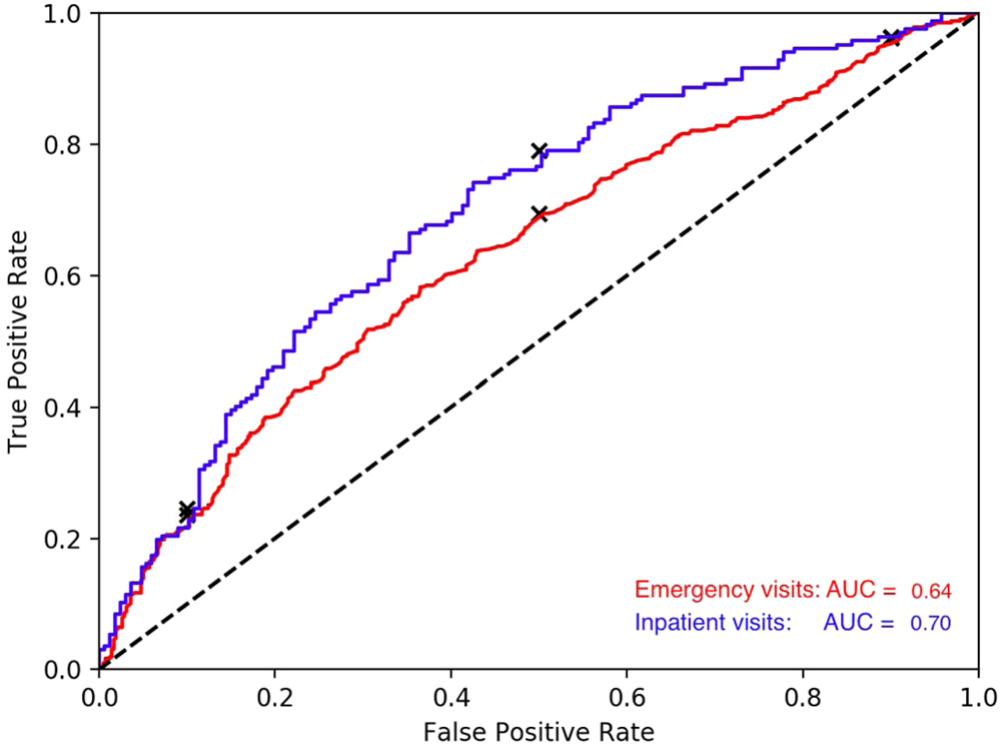
Area Under the Receiver Operating Curves of linear ensemble models forecasting emergency (ED) and inpatient visits. Black dots indicate sensitivity at specific false positive rates (10%, 50% and 90%). Black dashed line represents AUC of 0.5. Blue line indicates Inpatient visits and red line indicates emergency visits.

Using the linear ensembling of machine learning models, language change prior to ED visits was predicted with an AUC of 0.64 (F1 score = 0.61) and those prior to inpatient visits with an AUC of 0.70 (F1 score = 0.65). Figure 2 shows the Area Under the Receiver Operating Curves with True Positive Rates (sensitivity) vs. False Positive Rates (1-specificity). While the ensemble model obtained the best performance, we also performed an active control analysis using a simple modeling technique (logistic regression with ridge penalization) and obtained AUC of 0.69 (F1 score = 0.62) and 0.63 (F1 score = 0.60) for predicting inpatient and emergency visits respectively. We ran the same ensemble model on another control set (for the same patients) independently and obtained similar results (ED: AUC of 0.63, F1 score = 0.60; inpatient: AUC of 0.69 and F1 score = 0.62). The methods we implemented achieved predictive power comparable to methods that relied on direct clinical information such as prior hospitalizations and primary outcomes for patients hospitalized for at least 24 hours^13–15^.

### Identifying differentially expressed language features prior to a hospital visit

Tables 2 and 3 show the differentially expressed language features prior to ED and inpatient visits respectively. Table 4 shows several representative examples of posts. All represented posts were de-identified and paraphrased for anonymity.

**Table 2:** Statistical insights on differential language expression prior to an emergency visit*.

Feature	Cohen’s d	p-value (corrected)	Mean diff-of-diff	95% CI
**Emergency**				
*Change in Linguistic Style/ Mental Well-being*				
*Categories that increase in usage before emergency visit*				
Anxious	0.241	<0.001	0.070	[0.1, 0.38]
Depressed	0.238	<0.001	0.066	[0.1, 0.37]
Arousal (how exciting the post is)	0.191	0.011	0.051	[0.06, 0.33]
Valence (positive affect)	0.168	0.017	0.079	[0.03, 0.3]
#posts b/w 9am-12pm	0.160	0.020	0.016	[0.02, 0.3]
#posts b/w 12-3 pm	0.144	0.047	0.013	[0.01, 0.28]
*Categories that decrease in usage before emergency visit*				
Netspeak (‘u’, ‘da’, ‘smh’)	−0.374	<0.001	−0.01	[−0.51, −0.24]
1st person singular (‘I’, ‘my’, ‘me’, ‘I’m’)	−0.345	<0.001	−0.012	[−0.48, −0.21]
Informal Speech (‘lol’, ‘:)’, ‘b’)	−0.345	<0.001	−0.012	[−0.48, −0.21]
Leisure (‘family’, ‘fun’, ‘play’, ‘nap’)	−0.225	0.001	−0.002	[−0.36, −0.09]
*Change in Linguistic Topics*				
*Topics that increase in usage before emergency visit*				
hospital, pain, surgery, blood, doctor, nurse	0.230	0.001	0.001	[0.09, 0.37]
kids, child, their, children, mother, father	0.165	0.021	<0.001	[0.03,0.3]
even, still, tho, though, yet, blah	0.165	0.021	<0.001	[0.03,0.3]
thankful, very, amazing, most, blessed, wonderful	0.142	0.013	<0.001	[0.01,0.28]
*Topics that decrease in usage before emergency visit*				
luv, nite, sum, 2day, kidz, doin	−0.315	<0.001	−0.001	[−0.45, −0.18]
<3, tht, lovin, bt, missin, ima	−0.308	<0.001	<0.001	[−0.44, −0.17]
nite, fb, bed, gn, sleep, night	−0.295	<0.001	−0.001	[−0.43, −0.15]
jus, bored, crib, house, chilin, hmu	−0.287	<0.001	−0.001	[−0.42, −0.14]

*Positive cohen’s d indicates an increase in the given style, while a negative score indicates decrease. Effect sizes of individual linguistic features (diff-of-diff b/w true and null events) for emergency visits. Significance was measured using paired, two-tailed t-test with Benjamini-Hochberg p-correction.

**Table 3:** Statistical insights on differential language expression prior to an inpatient visit*.

Feature	Cohen’s d	p-value (corrected)	Mean diff-of-diff	95% CI
**Inpatient visits**				
*Change in Linguistic Style/ Mental Well-being*				
Categories that increase in usage before inpatient visit				
Depressive	0.306	0.001	0.089	[0.09, 0.52]
Anxious	0.286	0.001	0.084	[0.07, 0.50]
Family (‘baby’, ‘ma’,‘son’, ‘family’)	0.306	0.001	0.003	[0.09, 0.52]
Health (‘tired’, ‘pain’, ‘sick’, ‘ill’)	0.255	0.032	0.001	[0.04, 0.48]
Categories that decrease in usage before inpatient visit				
1st person singular (‘lol’, ‘:)’, ‘b’)	−0.392	<0.001	−0.014	[−0.61, −0.17]
Informal Speech (‘lol’, ‘:)’, ‘b’)	−0.392	<0.001	−0.014	[−0.61, −0.17]
Hear (‘say’, ‘hear’, ‘listen’, ‘heard’)	−0.365	0.003	−0.001	[−0.58, −0.15]
Affiliation (‘we’, ‘our’, ‘friends’)	−0.361	0.001	−0.003	[−0.58, −0.14]
Extraverted	−0.357	<0.001	−0.080	[−0.57, −0.14]
Drives (‘up’, ‘get’, ‘love’)	−0.354	<0.001	−0.006	[−0.57, −0.14]
Netspeak (‘u’, ‘lol’, ‘da’, ‘smh’)	−0.35	<0.001	−0.01	[−0.57, −0.13]
Nonfluencies (‘ugg’, ‘well’, ‘oh’, ‘er’)	−0.335	0.001	−0.001	[−0.55, −0.12]
Leisure (‘fun’, ‘play’, ‘nap’)	−0.321	0.006	−0.002	[−0.54, −0.1]
Reward (‘get’, ‘take’, ‘best’, ‘win’)	−0.314	0.001	−0.002	[−0.53, −0.1]
Affective Processes (‘:)’, ‘ugh’, ‘happy’)	−0.242	0.023	−0.004	[−0.46, −0.03]
Positive Emotion (‘love’, ‘good’, ‘lol’, ‘better’)	−0.228	0.013	−0.004	[−0.44, −0.01]
Swear Words (‘a**’, ‘f**k’, ‘hell’, ‘wtf’)	−0.209	0.023	−0.002	[−0.43, 0.00]
*Change in Linguistic Topics*				
*Topics that increase in usage before inpatient visit*				
check, yes, doctors, office, waiting, appointment	0.504	<0.001	0.001	[0.29, 0.72]
hospital, pain, surgery, blood, meds, nurse	0.380	<0.001	0.001	[0.16, 0.6]
baby, mommy, girl, son, boy, daughter	0.377	<0.001	0.001	[0.16, 0.59]
days, more, two, weeks, until, couple	0.301	0.014	0.001	[0.08,0.52]
even, still, tho, yet, blah, mad	0.275	0.006	0.001	[0.06, 0.49]
kids, child, their, children, mother, father	0.275	0.006	0.001	[0.06,0.49]
hurt, head, bad, body, stomach,:(, ugh	0.267	0.026	0.001	[0.05, 0.48]
*Topics that decrease in usage before inpatient visit*				
calling, phone, answer, hear, ooo, talking	−0.488	<0.001	−0.001	[−0.71, −0.27]
better, feeling, little, hope, bit, type	−0.429	<0.001	−0.001	[−0.65, −0.21]
lol, ha, ctfu, lmao, funny, haha	−0.429	<0.001	−0.001	[−0.65, −0.21]
cool, funny, tho, used, remember, seem	−0.395	<0.001	<0.001	[−0.61, −0.18]
no, what, matter, how, always, end	−0.381	<0.001	<0.001	[−0.6, −0.16]
:), show, crew, awesome, fashion, guys	−0.381	<0.001	<0.001	[−0.6, −0.16]

*Effect sizes of individual linguistic features (diff-of-diff b/w true and null events) for inpatient visits. Significance was measured using paired, two-tailed t-test with Benjamini-Hochberg p-correction.

**Table 4:** Sample social media posts in the month prior to an inpatient or ED visit.

Encounter diagnosis	#days prior to encounter	Example post (redacted)
**Inpatient encounters**		
Delivery complicated by asthma with acute exacerbation	1	just…had a major asthma attack…been like this for 3 days n today was the worst
Heart failure exacerbation	1	…cant sleep:(…as soon as I get to lay on my stomach I gotta deal wit this
Angina in the setting of heart failure	25	At Wendy’s for dinner…to make it healthy, ordered water instead of coke to go with my cheeseburger and fries
Unipolar major depression with psychotic features	1	GOIN IN CIRCLES…ROUND AND ROUND…I FEEL SO STUPID FOOLISH LOVING U THIS WAY……the tears…the hurt…i wish that u would just appear
Hysterectomy	1	…I gotta go all day without food….So pissed off…I know I’m scheduled to have the surgery…tomorrow
Pelvic inflammatory disease	1	I’m so sick…I’m ready to go to the emergency room…it took me an hour to get up and pee…
**ED encounters**		
Spider bite	1	I was bit by something…my [arm] is purple and sore…I think it was a [bug]… I feel weak…if I’m here tomorrow I’ll go to [the ED]. I pray I don’t have [Lyme disease]…
Urinary tract infection	6	Woke up in…my own blood…totally anemic…I write this for 3 reasons: 1. awake and drinking [cranberry juice]….2….the past [4] months taught me that problematic…bladder and GI tract are unbelievably annoying….3….I love [worrying], attention, and lists
Breast pain (etiology unclear)	3	My heart is so heavy right now….R.I.P grandpa…
Nausea with vomiting (etiology unclear)	3	…juice: please stay in my body…I don’t want to reexperience it
Nausea with vomiting (etiology unclear)	2	So sick ughh cant take this. Im so bored and not feeln good at all
Panic attack	1	I stick my hand out for you…but you don’t give me a hand back…

Dictionary-based: Prior to ED visits, patients less more likely to post about *leisure* (d = −0.225), associated words such as ‘family’, ‘fun’, ‘play’, ‘nap’, internet slang (*netspeak)* (d = −0.374) words such as ‘:)’, ‘fb’, ‘ya’, ‘ur’, and *informal* language (d = −0.345) with words such as ‘u’, ‘lol’, ‘smh’, ‘da’. Patients also use *personal pronouns* less (d = −0.345) prior to ED visits compared to random time windows. In addition to these, prior to inpatient visits, patients increasingly post about *family* (d = 0.306) using words such as ‘baby’, ‘ma’, ‘son’; *health* (d = 0.255) using words such as ‘tired’, ‘pain’, ‘sick’, ‘ill’; and post less about their *drives* (d = -0.354) with words such as ‘up’, ‘get’, ‘love’, ‘good’, use less swear words (d = -0.209), less non fluences (d = -0.335) such as ‘ugg’, ‘oh’, ‘er’ and less positive emotion (d = -0.228) words such as ‘love’, ‘good’, ‘better, and *affiliations* (d = -0.361) with words such as ‘we’, ‘our’, ‘friends’.

Open-vocabulary: Posts prior to ED visits had an increased usage of topics related to *family* (d = 0.165, ‘kids’, ‘child’, ‘their’, ‘mother’) and words indicative of *hospital visits* (d = 0.230, ‘hospital’, ‘paid’, ‘blood’, ‘doctor’), and gratitude (d = 0.142, ‘thankful’, ‘amazing’, ‘blessed’). In addition to these, prior to inpatient visits, patients increasingly post about doctor appointments (d = 0.504, ‘check’, ‘yes’, ‘doctor’, ‘office’, ‘waiting’) and somatic issues (d = 0.267, ‘hurt’, ‘head’, ‘bad’, ‘body’, ‘stomach’).

Mental well-being: Anxious and depressed language increased significantly (d = 0.241 and 0.238, respectively) prior to an ED visit. Increases were found in positive valence (d = 0.168) and arousal (d = 0.160) as revealed by users’ language. Also, the frequency of posts between 9am-3pm increased prior to an ED visit. Language changes prior to inpatient visits had higher effect sizes on several of these categories. Depressed and anxious language increased significantly (effect size, d = 0.306 and 0.286 respectively), and increase in extroverted language was more prominent (d= −0.357) during random time windows compared to that prior an inpatient visit.

Posts in context: The examples in Table 4 demonstrate that the words used in posts may also provide insights about patients’ health related behaviors, symptoms, and intentions to present to the hospital. Health related behaviors could be risk factors - one patient reported eating a “cheeseburger and fries” 25 days prior to an admission for angina in the setting of heart failure – or steps leading to procedure - another patient posted confirming that they were fasting in the 24-hour lead up to a scheduled surgery. Symptoms were sometimes specific, like a patient complaining of being unable to sleep when lying flat, consistent with orthopnea, one day prior to an admission for a heart failure exacerbation. At other times, they were more vague, like a patient describing a “heavy heart” in the setting of the passing of a grandparent 3 days prior to an ED visit for “breast pain”, or a post describing feeling “sick” and “so bored” 2 days before an ED visit for nausea of unclear etiology. Patients often gave warning prior to presenting to the hospital: one patient wrote, “I’m so sick…I’m ready to go to the emergency room…it took me an hour to get up and pee” one day before an inpatient admission for pelvic inflammatory disease.

## Discussion

This study has three main findings. First, people use different language in their Facebook posts prior to an ED visit or inpatient admission compared to other times. Second, a machine learning model based on these language differences distinguished ED and inpatient visit months from non-visit month with AUCs of 0.64 and 0.70 respectively. Third, a machine learning model identified linguistic markers that are differentially expressed prior to hospital visits. One of the salient themes that emerged the month prior to ED and inpatient visits was that anxious and depressed language increased significantly. Similar associations have been found with psychological stress^16,17^, quality of life and physical health in general^18^. A manual review of posts showed that posts often describe specific risk factors and symptoms that align with an eventual diagnoses. Collectively, these results suggest that analyzing non-clinical data from social media posts has the potential to forecast hospital utilization for certain conditions.

Prior efforts to predict healthcare visits have relied on clinical information such as prior hospitalizations and primary outcomes for patients hospitalized for at least 24 hours. Such features were found to predict 30-day readmission risk using deep learning models with an AUC of 0.75^13^. Also, prediction of readmission risk post hospitalizations for heart failure did not seem to be improved by using self-reported socioeconomic, health status, and psychosocial characteristics (to an AUC of 0.65)^14^. While prediction of health care utilization has been examined in terms of readmission for specific diseases^15^, researchers are investigating non-traditional sources to supplement clinical and sociodemographic information that give insight into everyday aspects of one’s life in their natural environment^19^. These ecological factors could potentially complement information directly obtained in a clinic or laboratory. Other work using web search behavior suggests that it is possible to predict future patient visits from geotagged mobile search logs^20^, though they provide little insight on the context of healthcare utilization. The findings in this study show promise to use social media in not only forecasting hospital visits at the patient level but also gaining insight into patients’ behavior.

One-fifth of the world’s population uses Facebook and Twitter^21^, and people are increasingly sharing information about their health on social media sites^22^. Social media has been used to study a wide variety of health-related outcomes including depression^4^, stress^17^, and schizophrenia^23^. The benefits of studying patterns of language use as opposed to other sources of “big data” is that words are more easily interpretable, enabling studies to not only test the predictive power of social media but also to generate insights.

Utilizing nontraditional digital sources may allow clinicians to intervene earlier and design interventions to target patients at higher risk of readmission or excessive hospital utilization. In future studies, machine learning models could target specific patient populations to alert clinicians when there are patients at higher risk of readmission before the event occurs. The clinician could then intervene by addressing the patient’s medical needs with the goal of preventing the hospital visit. For instance, a patient complaining online of persistent fever despite antibiotic therapy could be flagged for more immediate follow-up, or a patient reporting doubts about what, if anything, could be eaten during bowel preparation prior to a surgery could be contacted proactively with an automated message outlining basic instructions.

This study has several limitations. First, although the demographics of our sample are similar to the overall population served by ED of our health system, our sample is not representative of the general population. Our sample represents both a historically underserved and non-representative sample. This was a convenience sample of patients receiving care at an academic, urban health system who indicated they used social media and were willing to share access to their EMR and social media data. Second, the EMR data about visits is obtained from a single health system whereas patients might have received care from other systems which are not captured in our analysis. Third, language use is highly regional and so the particular terms, or even the patterns of use, modeled in one region may not well predict events in another.

The potential to glean such personal information about individuals when they might visit the hospital from social media reveal challenges associated with addressing several ethical and privacy concerns. Research has found that a large proportion of patients are willing to securely share their personal data sources^24^ and are open to linking it to their electronic health record^22^ for research purposes. In this type of work, privacy is of central importance. When consenting patients, we stress that their clinicians are not surveilling their Facebook posts directly; rather, the de-identified data are automatically processed in aggregate and knowledge is drawn from the data to help us learn how to better address patient needs. Transparency about how, why and by whom these health indicators are used is critical. A multi-disciplinary approach of involving clinicians, computer scientists, policy makers, ethicists can inform how to analyze and integrate predictive models based on digital traces into systems of care^25^.

## Methods

This study was approved by the University of Pennsylvania Institutional Review Board. De-identified data necessary to reproduce the results contained in the document are available upon request. We will not, however, share individual-level Facebook data as it contains potentially identifying information about patients enrolled in the study. We will not share any EMR data. Data from participants was obtained from all participants with informed consent. All methods were performed and results presented in accordance with HIPAA guidelines.

### Participants

The social mediome study^26^ is an ongoing study of consenting patients who share access to their social media and EMR data. Using a convenience sample framework, patients receiving care in an urban academic hospital system are approached for study participation. Patients who consent to share their Facebook posts and access to their EMR are enrolled. We do not access data from study participants’ friends or from posts on the study participants’ pages made by anyone other than the participant. Additional details about the study design are described elsewhere^26^. For each participant we collect data from the EMR regarding demographics (age, race, gender), date and primary reason for every ED visit and inpatient admission.

### Study design

We used the case-crossover design to distinguish change in a user’s social media language features before a hospital visit compared to the change prior to any random time point (Fig. 1). In case-crossover studies, the existence of triggers before an event are compared to the existence of triggers at prior time periods from the same participant, allowing a more precise estimate of the association of triggers with events because each patient serves as his or her own control. We first describe how different time periods were defined for comparison and then describe the approaches we used to quantify features from language.

### Time periods before a hospital visit and random time points: defining true and null events

Using the EMR, we identified emergency and inpatient visits for all participants who consented to share their social media data. Using the case-control design, two periods were defined per person: one 2.5 month period before a hospital visit (true event–the “case”), and one 2.5 month period before a random date (null event–the “control”). For the *case*, we selected the most recent visit date for each participant with more than 20 posts in each month. The users meeting this inclusion criteria were used for subsequent analysis. The day of the hospital visit was removed, and only the messages prior to that date were used in the analysis.

To control for time-invariant differences between individuals (e.g. the use of hashtags was constantly increasing in time) we made predictions based on change in language rather than direct language use. Within each 2.5 month period, change was defined as the difference between two 30 day periods, separated by 15 days (see Fig. 1). We compared age, gender, and race of those who met the inclusion criteria versus others and found similar distribution. Based on prior research^27^, we chose 20 posts as the threshold within each window, and consequently we used 30 days as the window length and the gap between both windows was set to 15 days for emergency and inpatient settings.

In this longitudinal analysis, every patient is his or her own control–i.e. language prior to the null event is considered as control and language prior to the true event is considered part of the case group.

### Defining language features

We use three sets of linguistic features for measuring change in language: a) open-vocabulary topics^28^, b) dictionary-based psycholinguistic features^29^, c) style features such as valence (positive or negative affectivity), arousal (how calming or exciting the post is)^30^, and extent of anxious, extraverted, and depressed language^31^ by applying previously developed statistical models, and meta features such as posting statistics (average number of 1-grams) and time of posts. These have been shown to be predictive of several health outcomes such as depression^4^, schizophrenia^32^, attention deficit hyperactivity disorder (ADHD)^33^, and general well-being^34^. As researchers have used various types of biomarkers for diseases^35,36^, our aim was to identify the language markers^37^ among these features that are predictive and are differentially expressed prior to hospital visits.

From each post, we extracted the relative frequency of single words and phrases (consisting of two or three consecutive words). The distribution of two hundred latent dirichlet allocation (LDA) open vocabulary topics generated from a different cohort (consisting of N = 999 further patients)^38^, was then extracted from each post. The distribution of Linguistic Inquiry Word Count (LIWC) dictionary features^39^ was also extracted for each post. We extracted the relative frequency of each attribute (i.e., the total number of times a word written by the user matches a word in a given attribute, divided by the user’s total number of words). After aggregating these features for every user in each time window, the difference was then calculated to measure language change prior to hospital visit and prior to a random time point.

Finally, in order to understand language trends by diagnosis, two coders, DCS and SCG, identified presumed primary diagnoses for inpatient and ED encounters based on the combination of provider-reported ICD codes. DCS and SCG then reviewed Facebook posts prior to these encounters to identify representative examples. Examples were agreed upon by both coders and paraphrased in order to preserve sentiment while maintaining anonymity.

### Statistical analysis

#### Predicting hospital visits using changes in language prior to the visit

The aim of our prediction analysis was to assess whether a machine learning algorithm can automatically predict changes in linguistic markers prior to a hospital visit compared to a null event. For each patient, every hospital visit was paired with a null event.

Cross-Validation: The predictive model was tested using 5-fold cross-validation^40^, where the users in the training and test folds were mutually exclusive. Thus, the predictive model was created 5 times without any outcome information outside of the training data. A dimensionality reduction step (using Principal component analysis) was employed on all the language features to remove correlated features before input to the predictive model. We used linear ensembling (averaging) of several classifiers (Random Forest, Support Vector Machine, Gradient Boosting Machine and Logistic Regression) from the scikit-learn^41^ module in Python 3.4 language to fit all our models on the training folds. This is similar to the autoML paradigm of model selection which not only tends to give better predictive performance but also prevents any user bias from being introduced into the resulting model, such as a preference for one algorithm over another or prior knowledge about the dataset that can be exploited^42^. The ensemble trained on each training set was then applied on the corresponding held-out test set. This was repeated 5 times; the overall predictive performance is reported in terms of the Area Under the ROC Curve (AUC) and F1-score.

#### Identifying differentially expressed language features prior to a hospital visit

Within each participant, we then compared all the features from the windows prior to true and null events using a two-tailed paired t-test (alpha = 0.05). We used Benjamini-Hochberg p-correction to control the False Discovery Rate and a threshold of p < 0.05. We measured the effect sizes using Cohen’s d.
