# Author Correction: Variability in Language used on Social Media Prior to Hospital Visits

**DOI:** 10.1038/s41598-020-68555-5

**Published:** 2020-07-07

**Authors:** Sharath Chandra Guntuku, H. Andrew Schwartz, Adarsh Kashyap, Jessica S. Gaulton, Daniel C. Stokes, David A. Asch, Lyle H. Ungar, Raina M. Merchant

**Affiliations:** 1 University of Pennsylvania, Philadelphia, PA USA; 2 Stony Brook University, New York, NY USA; 3 Children’s Hospital of Pennsylvania, Philadelphia, PA USA; 4 Cpl Michael J Crescenz VA Medical Center, Philadelphia, PA USA

Correction to:* Scientific Reports* 10.1038/s41598-020-60750-8, published online 12 March 2020

The Article contains errors.

In the Results section under the subheading “Identifying differentially expressed language features prior to a hospital visit”

 “Dictionary-based: Prior to ED visits, patients less more likely to post about leisure (d =  − 0.225), associated words such as ‘family’, ‘fun’, ‘play’, ‘nap’, internet slang (netspeak) (d =  − 0.374) words such as ‘:)’, ‘fb’, ‘ya’, ‘ur’, and informal language (d =  − 0.345) with words such as ‘u’, ‘lol’, ‘smh’, ‘da’. Patients also use personal pronouns less (d =  − 0.345) prior to ED visits compared to random time windows.”

should read:

“Dictionary-based: Prior to ED visits, patients were less likely to post about leisure (d =  − 0.225) with words such as ‘fun’, ‘play’, ‘nap’, internet slang (netspeak) (d =  − 0.374) such as ‘u’, ‘da’, ‘smh’, and informal language (d =  − 0.345) with words such as ‘lol’, ‘:)’, ‘b’.”

In Table 2 under “Categories that decrease in usage before emergency visit”, the row “1st person singular” is a duplication of row “informal speech”.

In Table 2 under “Change in linguistic topics”, the row “even, still, tho, though, yet, blah” is a duplication of the row “kids, child, their, children, mother, father”.

The correct Table 2 appears below as Table 1

**Table 1:** Statistical insights on differential language expression prior to an emergency visit*.

Feature	Cohen's d	p value (corrected)	Mean diff-of-diff	95% CI
**Emergency**				
*Change in Lnguistic Style/Mental Well-being*				
*Categories that increase in usage before emergency visit*				
Anxious	0.241	< 0.001	0.070	[0.1, 0.38]
Depressed	0.238	< 0.001	0.066	[0.1, 0.37]
Arousal (how exciting the post is)	0.191	0.011	0.051	[0.06, 0.33]
Valence (positive affect)	0.168	0.017	0.079	[0.03, 0.3]
#posts b/w 9 am–12 pm	0.160	0.020	0.016	[0.02, 0.3]
#posts b/w 12–3 pm	0.144	0.047	0.013	[0.01, 0.28]
*Categories that decrease in usage before emergency visit*				
Netspeak (‘u’, ‘da’, ‘smh’)	− 0.374	< 0.001	− 0.01	[− 0.51, − 0.24]
Informal Speech (‘lol’, ‘:)’, ‘b’)	− 0.345	< 0.001	− 0.012	[− 0.48, − 0.21]
Leisure (‘fun’, ‘play’, ‘nap’)	− 0.225	0.001	− 0.002	[− 0.36, − 0.09]
*Change in Linguistic Topics*				
*Topics that increase in usage before emergency visit*				
Hospital, pain, surgery, blood, doctor, nurse	0.230	0.001	0.001	[0.09, 0.37]
Kids, child, their, children, mother, father	0.165	0.021	< 0.001	[0.03,0.3]
Thankful, very, amazing, most, blessed, wonderful	0.142	0.013	< 0.001	[0.01,0.28]
*Topics that decrease in usage before emergency visit*				
luv, nite, sum, 2 day, kidz, doin	− 0.315	< 0.001	− 0.001	[− 0.45, − 0.18]
< 3, tht, lovin, bt, missin, ima	− 0.308	< 0.001	< 0.001	[− 0.44, − 0.17]
nite, fb, bed, gn, sleep, night	− 0.295	< 0.001	− 0.001	[− 0.43, − 0.15]
jus, bored, crib, house, chilin, hmu	− 0.287	< 0.001	− 0.001	[− 0.42, − 0.14]
lol, ctfu, funny, too, yo, lmao	− 0.262	< 0.001	− 0.001	[− 0.4, − 0.13]

*Positive cohen’s d indicates an increase in the given style, while a negative score indicates decrease. Effect sizes of individual linguistic features (diff-of-diff b/w true and null events) for emergency visits. Significance was measured using paired, two-tailed t-test with Benjamini–Hochberg p-correction.

In Table 3 under “Categories that decrease in usage before inpatient visit”, the row “1st person singular (‘lol’, ‘:)’, ‘b’)” is a duplication of the row “Informal Speech (‘lol’, ‘:)’, ‘b’)”.

In Table 3 under “Change in Linguistic Topics”, the row “even, still, tho, yet, blah, mad” is a duplication of the row “kids, child, their, children, mother, father”.

In Table 3 under “Topics that decrease in usage before inpatient visit”, the row “better, feeling, little, hope, bit, type” is a duplication of the row “lol, ha, ctfu, lmao, funny, haha” and the row “no, what, matter, how, always, end” is a duplication of the row “:), show, crew, awesome, fashion, guys”.

The correct Table 3 appears below as Table 2

**Table 2:** Statistical insights on differential language expression prior to an inpatient visit*.

Feature	Cohen's d	p value (corrected)	Mean diff-of-diff	95% CI
**Inpatient visits**				
*Change in Linguistic Style/ Mental Well-being*				
Categories that increase in usage before inpatient visit				
Depressed	0.306	0.001	0.089	[0.09, 0.52]
Anxious	0.286	0.001	0.084	[0.07, 0.50]
Family (‘baby’, ‘ma’,‘son’, ‘family’)	0.306	0.001	0.003	[0.09, 0.52]
Health (‘tired’, ‘pain’, ‘sick’, ‘ill’)	0.255	0.032	0.001	[0.04, 0.48]
Categories that decrease in usage before inpatient visit				
Informal Speech (‘lol’, ‘:)’, ‘b’)	− 0.392	< 0.001	− 0.014	[− 0.61, − 0.17]
Hear (‘say’, ‘hear’, ‘listen’, ‘heard’)	− 0.365	0.003	− 0.001	[− 0.58, − 0.15]
Affiliation (‘we’, ‘our’, ‘friends’)	− 0.361	0.001	− 0.003	[− 0.58, − 0.14]
Extraverted	− 0.357	< 0.001	− 0.080	[− 0.57, − 0.14]
Drives (‘up’, ‘get’, ‘love’, ‘good’)	− 0.354	< 0.001	− 0.006	[− 0.57, − 0.14]
Netspeak (‘u’, ‘lol’, ‘da’, ‘smh’)	− 0.35	< 0.001	− 0.01	[− 0.57, − 0.13]
Nonfluencies (‘ugg’, ‘well’, ‘oh’, ‘er’)	− 0.335	0.001	− 0.001	[− 0.55, − 0.12]
Leisure (‘fun’, ‘play’, ‘nap’)	− 0.321	0.006	− 0.002	[− 0.54, − 0.1]
Reward (‘get’, ‘take’, ‘best’, ‘win’)	− 0.314	0.001	− 0.002	[− 0.53, − 0.1]
Affective processes (‘:)’, ‘ugh’, ‘happy’)	− 0.242	0.023	− 0.004	[− 0.46, − 0.03]
Positive emotion (‘love’, ‘good’, ‘better’)	− 0.228	0.013	− 0.004	[− 0.44, − 0.01]
Swear words (‘a**’, ‘f**k’, ‘hell’, ‘wtf’)	− 0.209	0.023	− 0.002	[− 0.43, 0.00]
*Change in Linguistic Topics*				
*Topics that increase in usage before inpatient visit*				
Check, yes, doctors, office, waiting, appointment	0.504	< 0.001	0.001	[0.29, 0.72]
Hospital, pain, surgery, blood, meds, nurse	0.380	< 0.001	0.001	[0.16, 0.6]
Baby, mommy, girl, son, boy, daughter	0.377	< 0.001	0.001	[0.16, 0.59]
Days, more, two, weeks, until, couple	0.301	0.014	0.001	[0.08,0.52]
Kids, child, their, children, mother, father	0.275	0.006	0.001	[0.06,0.49]
Hurt, head, bad, body, stomach, :(, ugh	0.267	0.026	0.001	[0.05, 0.48]
*Topics that decrease in usage before inpatient visit*				
Calling, phone, answer, hear, ooo, talking	− 0.488	< 0.001	− 0.001	[− 0.71, − 0.27]
Lol, ha, ctfu, lmao, funny, haha	− 0.429	< 0.001	− 0.001	[− 0.65, − 0.21]
Cool, funny, tho, used, remember, seem	− 0.395	< 0.001	< 0.001	[− 0.61, − 0.18]
:), show, crew, awesome, fashion, guys	− 0.381	< 0.001	< 0.001	[− 0.6, − 0.16]
Wat, tht, juss, imma, luv, sum	− 0.366	< 0.001	< 0.001	[− 0.59, − 0.16]

*Effect sizes of individual linguistic features (diff-of-diff b/w true and null events) for inpatient visits. Significance was measured using paired, two-tailed t-test with Benjamini–Hochberg p-correction.

These corrections do not affect the conclusions of the Article.

